# Effects of Obstructive Sleep Apnea on Cognition: An Analysis of Electroencephalography Microstates and Alpha Peak Frequency

**DOI:** 10.1111/cns.70553

**Published:** 2025-08-06

**Authors:** Yan Wang, Ling Luo, Peilin Huang, Shaofan Jiang, Xiaodong Pan

**Affiliations:** ^1^ Department of Neurology Center for Cognitive Neurology, Fujian Medical University Union Hospital Fuzhou China; ^2^ Department of Neurology Quanzhou First Hospital of Fujian Medical University Quanzhou Fujian China; ^3^ Fujian Institute of Geriatrics Fujian Medical University Union Hospital Fuzhou China; ^4^ Institute of Clinical Neurology Fujian Medical University Fuzhou China

**Keywords:** biomarkers, cognitive impairment, EEG microstates, obstructive sleep apnea syndrome (OSA)

## Abstract

**Background:**

Respiration‐related sleep disorders such as obstructive sleep apnea (OSA) are risk factors for mild cognitive impairment and age‐related neurocognitive deficits. Nearly 60% of patients with OSA suffer from a variety of impaired cognitive functions, including attention, working memory, and episodic memory, and are susceptible to mood disorders such as depression and anxiety. However, it remains largely unexplored regarding the features of the electroencephalography (EEG) microstates of these patients and the potential association between the EEG microstates and the cognitive impairments.

**Method:**

In this study, we performed polysomnography (PSG) on 70 patients who were recruited from the Department of Neurology and subsequently categorized into the OSA group (44 patients) and the non‐OSA group (26 patients). We collected and analyzed their demographic information, blood and body fluid specimens, EEG alpha peak frequency, and EEG microstates.

**Result:**

Compared with the non‐OSA group, the OSA group reported more cases of comorbid anxiety symptoms (58.5% vs. 29%), and scored significantly lower in the word classification test. Of note, compared with the non‐OSA counterparts, the OSA patients displayed markedly more microstate A, a significantly higher rate of B to A microstate conversion, and drastically lower Delta and Theta power at the F7‐AV position.

**Conclusion:**

These findings confirm that OSA may affect sleep quality and cognitive function by influencing EEG activity and microstates, which highlights a potential EEG network mechanism for OSA‐induced cognitive impairment.

## Graphical abstract

In this study, we performed polysomnography (PSG) on 70 patients; findings confirm that OSA may affect sleep quality and cognitive function by influencing EEG activity and microstates, which highlights a potential EEG network mechanism for OSA‐induced cognitive impairment.

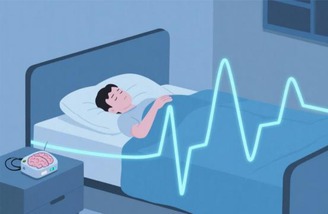

## 1 Background

Obstructive sleep apnea (OSA) is a sleep disorder that is characterized by recurrent episodes of upper airway obstruction, intermittent hypoxemia, and sleep fragmentation. The condition is prevalent in more than 55% of males and 30% of females over the age of 40 years [1]. Though a treatable disease, 80% of people with OSA remain undiagnosed and untreated [2]. Studies have demonstrated that patients with OSA run a significantly increased risk of cardiovascular events, including hypertension, congestive heart failure, stroke, myocardial infarction, etc. [3] and that OSA, if untreated, may induce excessive daytime sleepiness, resulting in daytime functional impairment and increased accident proneness [4]. Therefore, due attention should be steered to the timely screening and early diagnosis of OSA in the afflicted population.

OSA is closely linked to an array of clinical conditions. In patients with OSA, somnolence is a common clinical manifestation and exacerbates with the severity of OSA [5]. In addition to excessive daytime sleepiness, available evidence has associated OSA with significantly higher incidences of diabetes, kidney diseases, cardiovascular diseases, and cerebrovascular events [6]. In particular, the condition is prominently implicated in cognition‐related neuro‐deterioration. Studies show that OSA is independently associated with cognitive impairment [7, 8]. A recent survey reveals that compared with non‐OSA counterparts, patients with OSA report a significant increase in the number of cognitive impairment‐related symptoms (e.g., vigilance, concentration) [9]. Other studies have documented a host of comorbid cognitive impairment‐related symptoms, including memory loss, anxiety, depression, and decline in concentration and executive functioning [3, 8, 10, 11, 12, 13, 14, 15, 16, 17, 18, 19]. Still others suggest that OSA may be associated with earlier cognitive decline and with the progression of mild cognitive impairment or Alzheimer's disease (AD) [20]. Therefore, it is of great significance to explore the involvement and relevant mechanism of OSA in cognition‐related impairment.

Despite the unavailability of a clear elucidation of the cognition‐related deterioration in patients with OSA, some arguable notions have been suggested: the cleansing role of sleep, the impact of hypoxia, and the effect of sleep fragmentation. The former suggests that sleep may facilitate the clearance of metabolic products from the brain, removing toxic proteins and metabolic wastes accumulated during waking hours, including β‐amyloid and other substances that may be involved in the pathogenesis of dementia [21]. The latter two maintain that hypoxia during sleep and the severity of sleep fragmentation are closely related to the progression of OSA [22], leading to progressive destructive changes in brain structure and function, which make the brain more vulnerable to neurodegenerative diseases [23]. Depression may also be associated with obstructive sleep apnea syndrome, with those with obstructive sleep apnea syndrome being 2.4 times more likely to suffer from major depression than others [24]. However, recent literature has found that anxiety states may actually be more common than depressive states in severe cases of OSA. Some findings suggest that the severity of anxiety symptoms may depend on daytime sleepiness rather than nocturnal hypoxemia in patients with OSA [25]. More studies on OSA comorbidities are still needed, as they may have a stronger association with hypoxemia.

Currently, patients with OSA are routinely diagnosed by polysomnography (PSG), with the severity of OSA indicated by apnea hypoventilation index (AHI). On the scale, an AHI of < 5 breaths/h indicates a normal sleep; an AHI of 5–15 breaths/h suggests mild OSA; an AHI of 16–30 breaths/h, moderate OSA; and an AHI of > 30 breaths/h, severe OSA. Recently, electroencephalography (EEG) microstate analysis is adopted to capture the spatio‐temporal dynamics in patients with OSA to better depict their cognitive profiling. In this procedure, the global field power (GFP) of the EEG signal is calculated from the potential value of each electrode channel, and the peak GFP time series is extracted, from which four clustering centers—microstates A, B, C, and D (A: visual network; B: auditory network; C: salient and transformational network: D: attention network) are obtained by a clustering algorithm [26]. In the EEG microstate analysis of patients with AD, changes in the coverage of microstate C are correlated with the level of Aβ42 in the cerebrospinal fluid, while those of microstate B are correlated with the level of p‐tau protein [25]. As cognitive dysfunction worsens, the coverage of microstates A and B significantly increases, and that of microstates C and D decreases in a gradient‐like manner [27]. It follows that EEG microstates may be potential biomarkers for exploring early electrophysiologic abnormalities in α‐synucleinopathy. However, it remains unexplored regarding the characteristics of EEG microstates in patients with OSA.

In the current study, patients with OSA were enrolled with PSG as the diagnostic standard. The proportion of microstates A, B, C, and D and their mutual transformation was analyzed; the cognitive performance of the patients was assessed by routine cognition assessment scales; and the potential associations of OSA with changes in cognitive functions and EEG microstates were explored. The findings may highlight the clinical significance of EEG microstates in the treatment of OSA.

## 2 Methods

### 2.1 Subjects

Participants were recruited from patients who were admitted into the Department of Neurology of Union Hospital of Fujian Medical University from January 1, 2024, to June 30, 2024. On the basis of clinical manifestations and PSG results, the qualified participants were divided into the OSA group and the non‐OSA group (control). The inclusion criteria were as follows: age of 30–80 years and OSA diagnosed according to the criteria established by DSM‐V and ICSD. Informed consent was obtained from all the enrolled patients.

**FIGURE 1:**
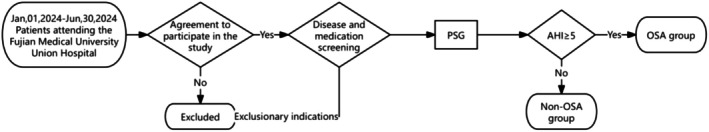
Flowchart of the admission process.

Patients were excluded from the study if they met any of the following criteria: diagnoses of non‐neurodegenerative CNS diseases (e.g., cerebrovascular diseases, infectious diseases, demyelinating diseases, trauma, tumors, etc.); comorbidities of other neuropsychiatric conditions; the use of medications for other medical conditions that may affect cognition, level of arousal, and quality of sleep; cooperative difficulty; or pregnancy. The enrollment process is displayed in Figure 1.

### 2.2 Subject Grouping

*OSA group*: Patients with PSG‐confirmed OSA.

*Non‐OSA group*: Patients without PSG‐confirmed OSA.

### 2.3 Observation and Collection Indexes

After the enrollment, an array of indices was collected. The basic information of each patient was recorded, including gender, age, BMI, residence, education/occupation type, disease duration, medication history, complications, etc.

The overall cognitive performance of all patients was assessed with various cognitive scales: overall cognitive function (MMSE, MoCA), attention, executive functions (Connectedness Test A, Connectedness Test B), verbal function (word fluency, Boston Naming Test), memory (Auditory Word Learning Test: Immediate Memory, Delayed Memory 1, Delayed Memory 2, Word Categorization, and Recognition Memory), visuospatial functioning (Drawing the Clock), mood evaluation (Hamilton Anxiety Scale, Hamilton Depression Scale), functional assessment (ADL score, IADL), and dementia level score (CDR score).

The sleep quality and performance were evaluated with the sleep scales: the Pittsburgh Sleep Quality Index (PSQI), Insomnia Severity Index (ISI), Epworth Sleepiness Scale (ESS), Chalder 14‐item Fatigue Scale, IRLSSG Restless Legs Syndrome (RLS) Severity Self‐Assessment Scale, and RBD Severity Questionnaire.

The sleep structures and stages were examined by polysomnography (PSG) and PSG‐related indices were collected, including total sleep time, sleep efficiency, number of awakening transitions, time awake after sleep onset, number of sleep staging transitions, sleep onset latency, REM latency, percentage of stage N1, percentage of stage N2, percentage of stage N3, percentage of stage REM, periodic limb movements during wakefulness, periodic limb movements during sleep, periodic limb movements related to microarousals, total microarousal index, apnea hypoventilation index, REM mean heart rate, NREM mean heart rate.

The EEG microstate analysis was performed by analyzing the related indices, including the duration, coverage, occurrence frequency, and transition probability of the microstates (A, B, C, and D). Moreover, multimodal heart‐brain electrophysiologic indices were collected: alpha‐shock peak frequency and resting‐state heart rate variability.

### 2.4 Data Management and Analysis

The data collected were processed and analyzed with R language (version 4.3.3). For continuous variables, data with a normal distribution were expressed as mean ± standard deviation; the inter‐group comparison was assessed by independent samples *t*‐test, and the association between OSA and cognition was processed by Pearson correlation analysis. For data without a normal distribution, they were described as median [P25, P75]; the inter‐group comparison was analyzed by two independent samples rank‐sum test; the correlation was processed by Spearman method; and the validation was by the FDR correction method. The counting data was expressed as the number of cases (%) and the inter‐group comparison was conducted by chi‐squared test and by Fisher's exact probability if the chi‐squared test was not satisfied. The box‐and‐line plots and correlation heat maps were depicted with the ggplot2 package. All tests were two‐sided, with the statistical difference set at *p* < 0.05.

## 3 Results

### 3.1 Basic Information

We collected 70 patients who were admitted to the cognitive ward of the Union Hospital of Fujian Medical University to complete polysomnographic monitoring from January 1, 2024, to June 30, 2024, including 44 patients in the OSA group and 26 patients in the non‐OSA group. The baseline data of the two groups are summarized in Table 1. Sex, age, body mass index (BMI), educational background, occupation, tobacco and alcohol intake, hypertension (HT), and diabetes mellitus (DM) were used as categorical variables. The inter‐group difference in these variables was analyzed by the chi‐squared test or Fisher's exact probability method. No statistical difference was found between the two groups.

**TABLE 1:** Basic information.

	Total (*n* = 70)	Non‐OSA (*n* = 26)	OSA (*n* = 44)	Statistic	*p*
Sex, *n* (%)				1.568	0.211
Man	31 (44.29)	9 (34.62)	22 (50.00)		
Woman	39 (55.71)	17 (65.38)	22 (50.00)		
Year, *n* (%)				Fisher	0.177
< 60	20 (28.57)	11 (42.31)	9 (20.45)		
60–80	45 (64.29)	14 (53.85)	31 (70.45)		
> 80	5 (7.14)	1 (3.85)	4 (9.09)		
BMI, *n* (%)				Fisher	0.130
< 24.9	41 (58.57)	19 (73.08)	22 (50.00)		
25–30	26 (37.14)	7 (26.92)	19 (43.18)		
> 30	3 (4.29)	0 (0.00)	3 (6.82)		
Residence, *n* (%)				Fisher	0.468
Alone	9 (12.86)	2 (7.69)	7 (15.91)		
Spouse	61 (87.14)	24 (92.31)	37 (84.09)		
Educational background, *n* (%)				Fisher	0.514
Illiteracy	16 (22.86)	7 (26.92)	9 (20.45)		
Elementary	13 (18.57)	3 (11.54)	10 (22.73)		
Above middle	41 (58.57)	16 (61.54)	25 (56.82)		
Occupation, *n* (%)				1.251	0.263
Physical	37 (52.86)	16 (61.54)	21 (47.73)		
Mental	33 (47.14)	10 (38.46)	23 (52.27)		
Smoking, *n* (%)				Fisher	0.664
No	64 (91.43)	23 (88.46)	41 (93.18)		
Yes	6 (8.57)	3 (11.54)	3 (6.82)		
Alcohol intake, *n* (%)				Fisher	1.000
No	69 (98.57)	26 (100.00)	43 (97.73)		
Yes	1 (1.43)	0 (0.00)	1 (2.27)		
HT, *n* (%)				2.369	0.124
No	43 (61.43)	19 (73.08)	24 (54.55)		
Yes	27 (38.57)	7 (26.92)	20 (45.45)		
DM, *n* (%)				Fisher	0.751
No	58 (82.86)	21 (80.77)	37 (84.09)		
Yes	12 (17.14)	5 (19.23)	7 (15.91)		

### 3.2 Cognitive‐Psychological Assessment

The cognitive scores of OSA and non‐OSA groups were analyzed for distributional normality, with the median and quartiles of each score of the two groups detailed in Table 2. The differences in the cognitive scores of MMSE, MoCA, and CDR were not statistically significant between the two groups, which is probably due to the small sample size and the homogeneity in the subjects who were admitted to the cognitive wards for similar conditions. However, the quartile analysis revealed less dispersion in the OSA group and more variability in the non‐OSA group. Of note, the OSA group fared worse on the three memory‐related scales, with the non‐OSA group reporting a significantly higher median score in the word categorization test than the OSA group (*U* = 2.087, *p* = 0.037). This result indicates that OSA may exacerbate cognitive deterioration, especially memory loss and executive dysfunction.

**TABLE 2:** Cognitive‐psychological assessment.

Variables	Total (*n* = 70)	No‐OSA (*n* = 26)	OSA (*n* = 44)	Statistic	*p*
MMSE, Median (Q1, Q3)	26.00 (21.00, 28.00)	25.00 (20.25, 28.00)	26.00 (22.50, 28.00)	−0.543	0.587
MoCA, Median (Q1, Q3)	20.00 (15.25, 24.00)	20.50 (13.00, 26.00)	20.00 (16.75, 23.25)	0.152	0.879
LigatureA, Median (Q1, Q3)	25.00 (25.00, 25.00)	25.00 (15.25, 25.00)	25.00 (25.00, 25.00)	−1.175	0.240
LigatureB, Median (Q1, Q3)	25.00 (3.00, 25.00)	25.00 (0.00, 25.00)	25.00 (12.00, 25.00)	−0.736	0.462
Word fluency, Median (Q1, Q3)	32.50 (25.25, 37.75)	30.50 (22.00, 43.75)	33.00 (26.00, 37.25)	−0.584	0.559
Named, Median (Q1, Q3)	22.00 (18.25, 25.75)	23.50 (20.25, 26.00)	22.00 (17.75, 24.25)	1.481	0.139
Instant memory, Median (Q1, Q3)	19.00 (12.25, 28.00)	22.00 (13.25, 30.50)	18.50 (11.75, 25.00)	0.973	0.330
Delayed memory1, Median (Q1, Q3)	5.00 (2.00, 7.00)	6.00 (2.25, 8.75)	5.00 (2.00, 6.25)	1.424	0.155
Delayed memory2, Median (Q1, Q3)	3.00 (1.00, 5.00)	3.00 (1.00, 6.75)	3.00 (0.75, 4.00)	1.028	0.304
Word classification, Median (Q1, Q3)	2.00 (0.00, 8.00)	9.00 (5.25, 9.75)	1.00 (0.00, 5.00)	2.087	0.037
Recertification, Median (Q1, Q3)	8.50 (6.00, 18.00)	8.00 (4.50, 15.75)	10.00 (6.00, 18.00)	−0.713	0.476
Draw clocks, Median (Q1, Q3)	2.00 (1.00, 3.00)	1.00 (1.00, 2.75)	2.00 (1.00, 3.00)	−0.894	0.371
ADL, Median (Q1, Q3)	8.00 (8.00, 8.00)	8.00 (8.00, 8.00)	8.00 (8.00, 8.00)	−0.634	0.526
CDR, Median (Q1, Q3)	0.50 (0.50, 1.00)	0.50 (0.50, 1.00)	0.50 (0.50, 0.62)	0.110	0.913

A significant difference in the prevalence of anxiety symptoms was evident between the non‐OSA group and the OSA group (29.2% vs. 58.5%, respectively; *U* = 5.234, *p* = 0.022). This indicates a high prevalence of anxiety in the OSA group, which may be related to the effect of OSA on emotion regulation and the need to incorporate psychological interventions in the treatment.

### 3.3 EEG Microstates

**FIGURE 2:**
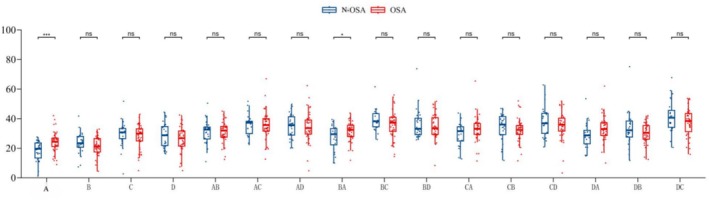
Correlation between OSA and EEG microstates. The lower edge of the box plot: First quartile (Q1, 25% quantile); the line in the middle of the box: Median (Q2, 50% quantile); the upper edge of the box: Third quartile (Q3, 75% quantile). As indicated, the upper whisker line extended to Q3 + 1.5 × IQR (interquartile spacing) or the maximum of the dataset (whichever is smaller); the lower whisker line extended to Q1 − 1.5 × IQR or the minimum value of the dataset (whichever is greater). Data points beyond the whisker line were considered outliers and indicated by a separate point. Significance of * and *** indicate the level of statistical significance. A single asterisk (*) represents *p* < 0.05, and three asterisks (***) represent *p* < 0.001.

The statistical results are shown in Table 3 and Figure 2. The overall median of microstate A was 22.44%, with 19.65% in the non‐OSA group and 24.38% in the OSA group (*p* = 0.001). The overall median of the conversion rate from state B to state A was 31.20%, with 29.50% in the non‐OSA group and 32.40% in the OSA group (*p* = 0.04).

**TABLE 3:** EEG microstates.

Variables	Total (*n* = 70)	No‐OSA (*n* = 26)	OSA (*n* = 44)	Statistic	*p*
A, Median (Q1, Q3)	22.44 (18.62, 25.96)	19.65 (13.39, 23.54)	24.38 (21.18, 26.85)	−3.269	0.001
B, Mean ± SD	22.33 ± 6.68	23.52 ± 7.24	21.63 ± 6.31	1.109	0.273
C, Median (Q1, Q3)	30.46 (25.13, 33.38)	30.76 (26.48, 33.38)	29.91 (24.95, 33.11)	0.869	0.385
D, Mean ± SD	26.93 ± 8.18	28.55 ± 8.12	25.98 ± 8.15	1.281	0.206
AB, Mean ± SD	30.94 ± 7.47	30.80 ± 8.21	31.02 ± 7.07	−0.113	0.910
AC, Median (Q1, Q3)	36.35 (31.38, 39.55)	37.30 (29.90, 38.20)	35.70 (31.60, 40.00)	−0.218	0.827
AD, Mean ± SD	34.41 ± 9.56	34.57 ± 8.47	34.31 ± 10.30	0.115	0.909
BA, Median (Q1, Q3)	31.20 (26.40, 34.20)	29.50 (22.30, 33.30)	32.40 (27.90, 35.50)	−2.055	0.040
BC, Median (Q1, Q3)	37.70 (32.45, 41.20)	38.20 (34.50, 43.40)	37.30 (31.30, 40.90)	1.382	0.167
BD, Median (Q1, Q3)	33.45 (29.25, 40.62)	33.15 (29.20, 40.30)	33.45 (29.35, 40.70)	−0.197	0.844
CA, Median (Q1, Q3)	32.50 (29.10, 35.82)	31.55 (24.92, 34.50)	33.10 (29.37, 36.70)	−1.460	0.144
CB, Mean ± SD	33.12 ± 8.56	34.39 ± 9.26	32.33 ± 8.11	0.932	0.356
CD, Mean ± SD	36.31 ± 9.59	37.90 ± 10.04	35.30 ± 9.27	1.061	0.294
DA, Mean ± SD	31.49 ± 9.05	28.71 ± 8.85	33.08 ± 8.88	−1.928	0.060
DB, Median (Q1, Q3)	30.75 (26.30, 36.52)	32.20 (27.55, 38.43)	30.45 (26.10, 35.35)	1.186	0.235
DC, Mean ± SD	38.88 ± 9.90	41.40 ± 10.79	37.34 ± 9.10	1.570	0.124

The data showed that compared with the non‐OSA group, the OSA group reported a significantly higher proportion of EEG microstate A and a higher conversion rate from microstate B to microstate A, suggesting that microstate A and B to A conversion rates may be related to the pathological state of OSA and may be a potential biomarker for the transformation of its brain network.

### 3.4 EEG Alpha Peak Frequency

**FIGURE 3:**
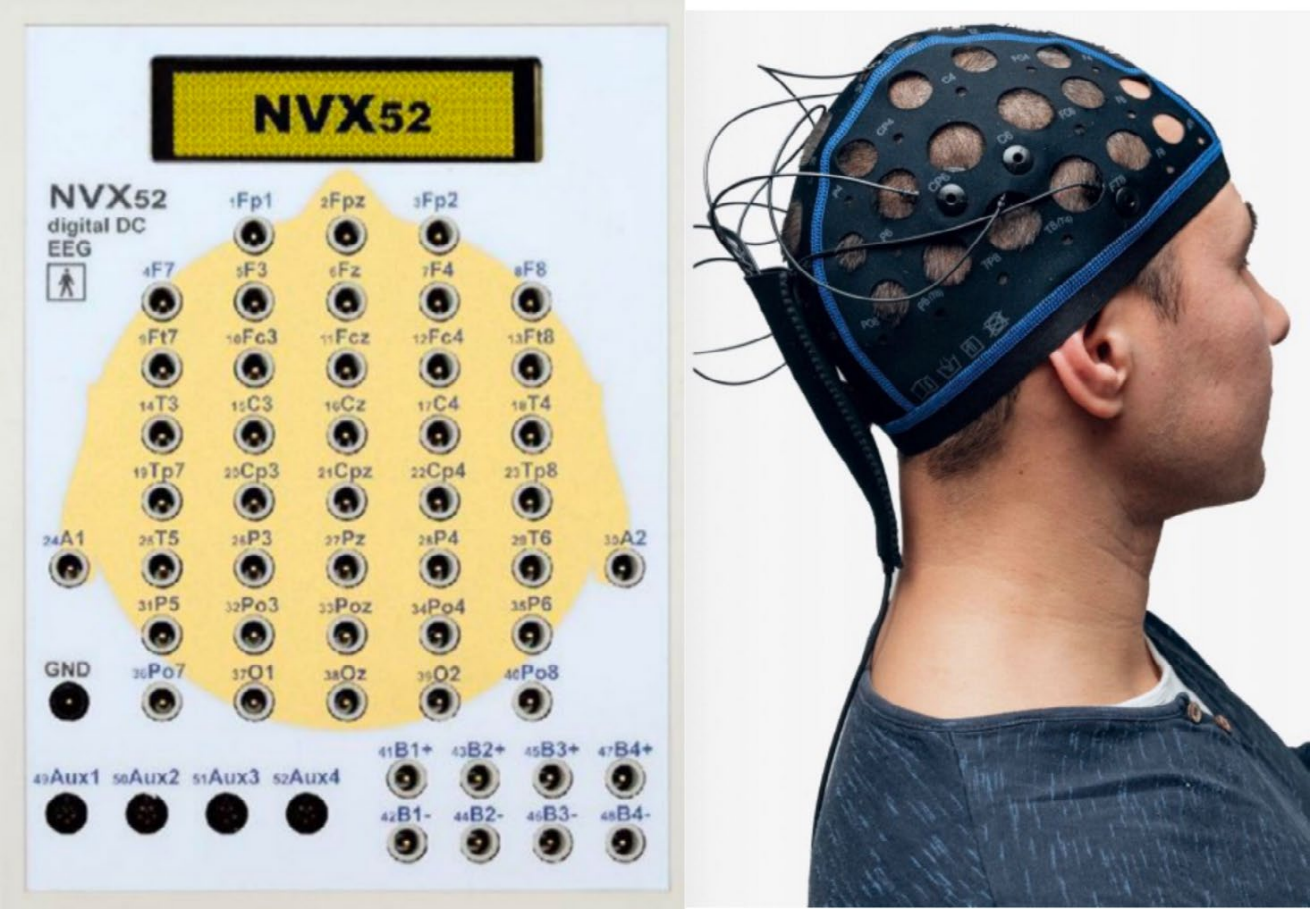
Schematic diagram of each lead of the EEG.

**FIGURE 4:**
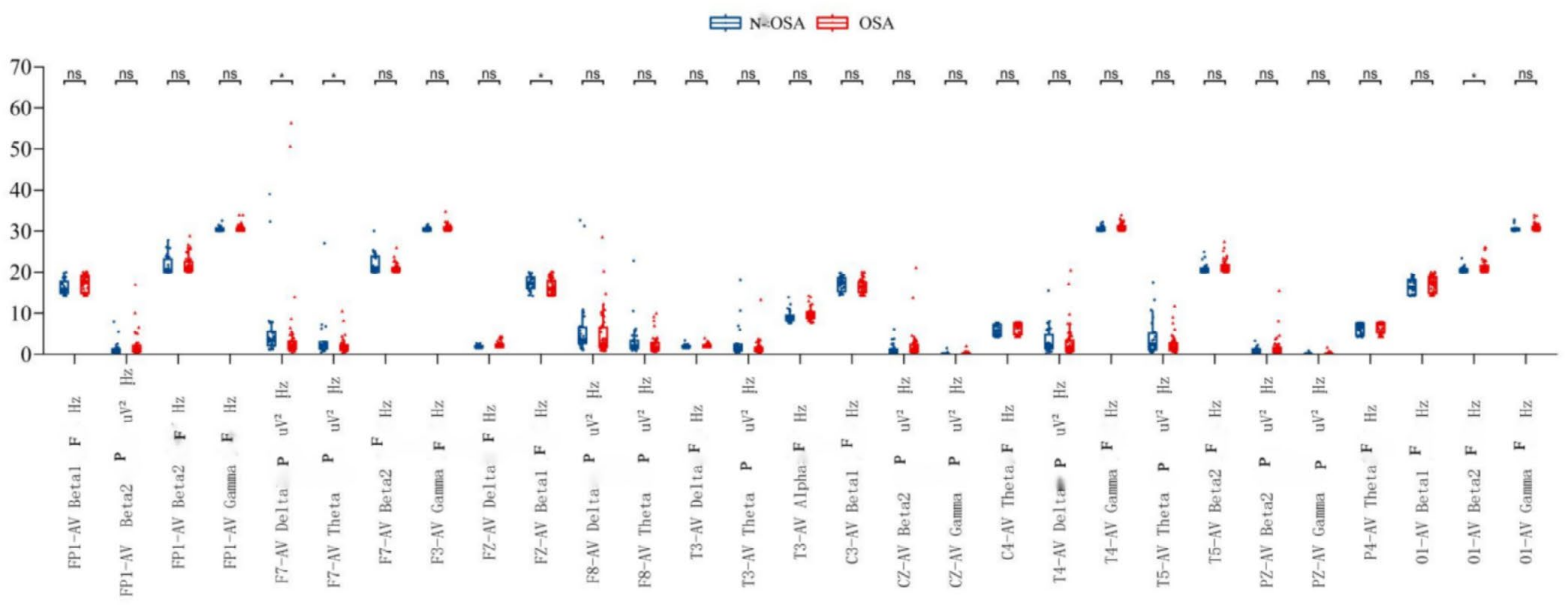
Correlation between OSA and EEG alpha peak frequency. The lower edge of the box plot: First quartile (Q1, 25% quantile); the line in the middle of the box: Median (Q2, 50% quantile); the upper edge of the box: Third quartile (Q3, 75% quantile). As indicated, the upper whisker line extended to Q3 + 1.5 × IQR (interquartile spacing) or the maximum of the dataset (whichever is smaller); the lower whisker line extended to Q1 − 1.5 × IQR or the minimum value of the dataset (whichever is greater). Data points beyond the whisker line were considered outliers and indicated by a separate point. Significance of * indicate the level of statistical significance. A single asterisk (*) represents *p* < 0.05.

The schematic diagram of the location of each lead of the EEG is indicated in Figure 3. The details of the EEG alpha peak frequency are shown in Figure 4. The results revealed significantly higher Delta and Theta power at the F7‐AV position in the non‐OSA group than in the OSA group (F7‐AV Delta power: 3.50 vs. 2.10, with an overall median of 2.45; *p* = 0.025; F7‐AV Theta power: 2.20 vs. 1.60, with an overall median of 1.75; *p* = 0.044). Moreover, compared with the OSA group, the non‐OSA group reported a higher Beta1 frequency at the FZ‐AV position (17.30 vs. 15.85, with an overall median of 16.50; *p* = 0.038). However, compared with the non‐OSA group, the OSA group reported a higher Beta2 frequency at the O1‐AV position (20.50 vs. 20.30, with an overall median of 20.50; *p* = 0.046).

### 3.5 Correlation Analysis of Cognition‐Sleep Parameters

**FIGURE 5:**
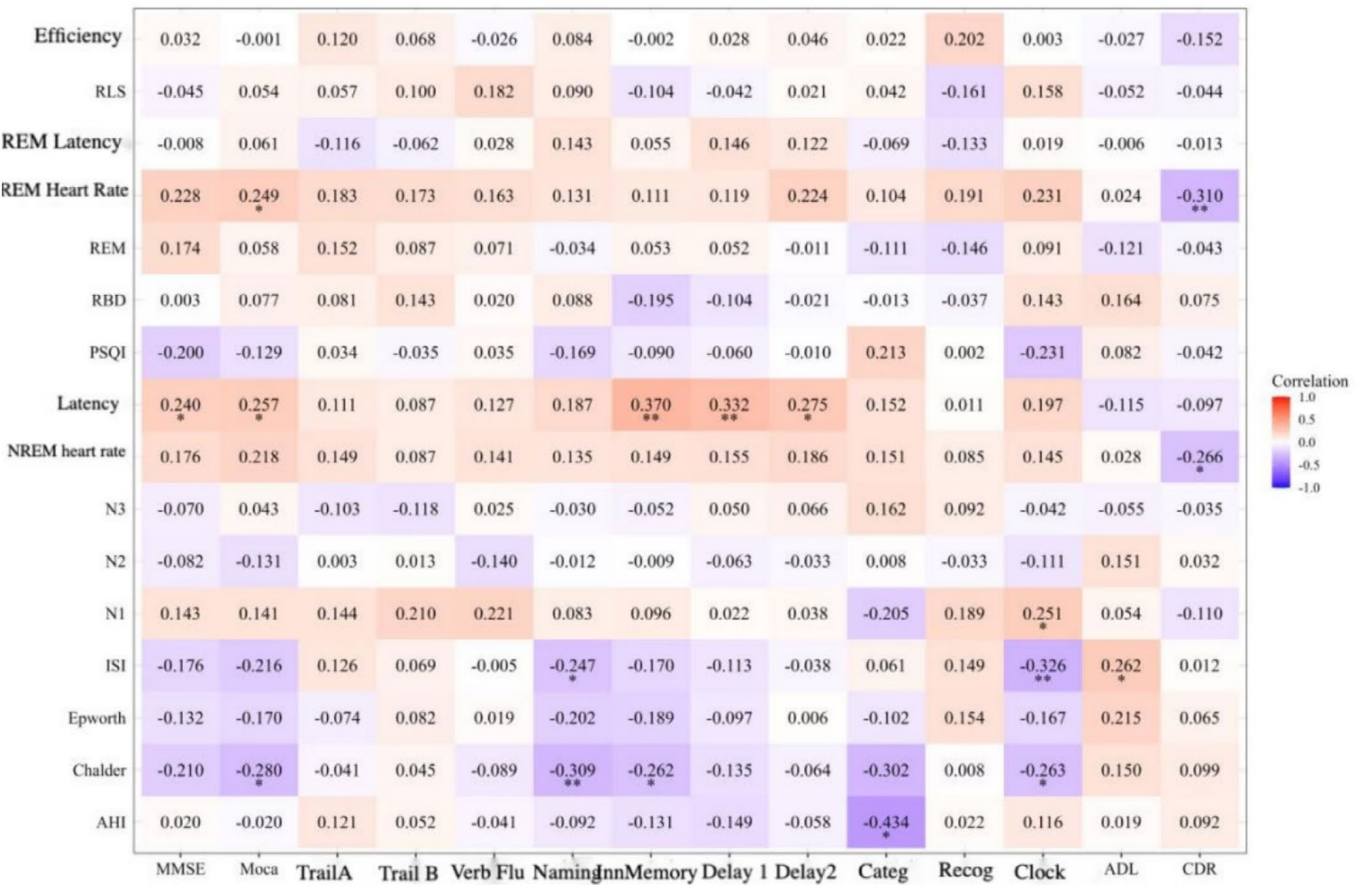
Correlation analysis of cognition‐sleep parameters. The color shades indicate the strength of the correlations, with darker colors signifying stronger correlations. The correlation coefficients in the graph range from −1 to 1, where 1 indicates a perfectly positive correlation and‐1 indicates a perfectly negative correlation. *p < 0.05, **p < 0.01.

The correlation between sleep parameters and cognitive function tests was depicted in the heat map (Figure 5). The strength of the correlations was indicated by the color shades, with darker colors signifying stronger correlations. The correlation coefficients in the graph ranged from −1 to 1, where 1 indicates a perfectly positive correlation and −1 indicates a perfectly negative correlation.

As shown in Figure 5, AHI was negatively correlated with word classification (−0.434); REM mean heart rate was positively correlated with MoCA (0.249) and negatively correlated with CDR (−0.310); PSG latency was positively correlated with MMSE (0.240), MoCA (0.257), immediate memory (0.370), latency 1 (0.332), and latency 2 (0.275); NREM mean heart rate was negatively correlated with CDR (−0.266); N1 was positively correlated with painted clock (0.251); ISI was negatively correlated with naming (−0.247) and painted clock (−0.326), and positively correlated with ADL (0.262); Chalder was positively correlated with MoCA (−0.280), naming (−0.309), immediate memory (−0.262), and word categorization (−0.302), and drawing clocks (−0.263). After FDR correction, a *p* value of ≤ 0.015 surfaced when the value of FDR was set at ≤ 0.05, so the statistical difference was only observed in the positive correlation between PSG latency and immediate and delayed memory, and in the negative correlation between AHI and word categorization.

### 3.6 Correlation Analysis of Cognition and EEG Microstates

**FIGURE 6:**
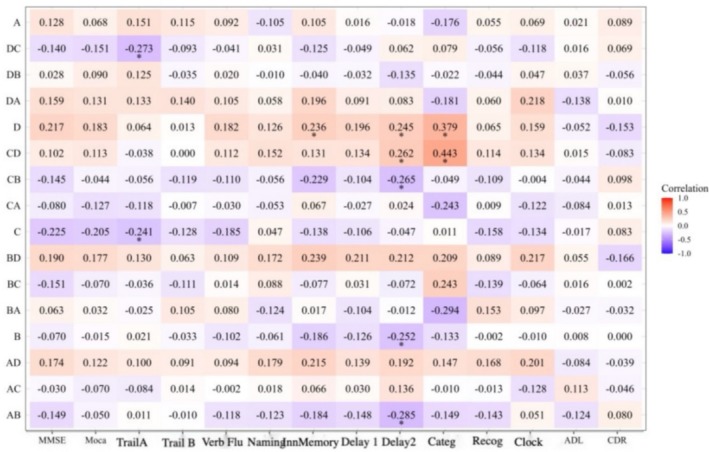
Correlation analysis of cognition and EEG microstates. As indicated, the double letter formation signifies the direction of the conversion, e.g., DC standing for the transition from state D to state C. *p < 0.05.

As shown in Figure 6, the conversion rate of D to C state transition was negatively correlated with the Link A task (−0.273); microstate D was positively correlated with immediate memory (0.236), delayed recall 2 (0.245), and word categorization (0.379); the conversion rate of C to D state transition was positively correlated with delayed recall 2 (0.262); word categorization (0.443), and the rate of C to B state transition was negatively correlated with delayed recall 2 (−0.265); microstate C was negatively correlated with linkage A (−0.241); microstate B was negatively correlated with delayed recall 2 (−0.252); and the rate of A to B state conversion was negatively correlated with delayed recall 2 (−0.285). Thus, microstate D (attention) and C to D conversion rate were positively correlated with word categorization and delayed recall. After FDR correction, a *p* value of ≤ 0.018 surfaced when the value of FDR was set at ≤ 0.05, so the statistical difference was only evident in the positive correlation between C to D state transition and delayed recall and in the negative correlation between A to B state transition and delayed recall.

## 4 Discussion

### 4.1 OSA and Cognitive‐Psychological Assessment

#### 4.1.1 Association Between OSA and Cognitive Impairment and Possible Mechanisms

In this study, the OSA group scored significantly lower in the word categorization test than the non‐OSA group, which was subsequently confirmed in the correlation analysis, suggesting that OSA may greatly impact cognitive function. This result is consistent with the findings of existent literature. A previous study suggests that OSA may severely impact the cognitive functioning, especially executive functioning, of the patients [28]. Another study documents that patients with OSA have significantly lower MoCA scores than healthy individuals, which is positively correlated with the AHI [29]. Available evidence indicates that patients with OSA are more prone to cognitive dysfunction if they are subjected to a hypoxic setting, which highlights that OSA screening should be prescribed for patients with cognitive decline [7]. The association of OSA with cognitive dysfunction has mainly been attributed to intermittent hypoxia (IH), sleep deprivation and fragmentation, hypercapnia, and neuroinflammation [30]. Studies have demonstrated that OSA‐induced hypoxemia can trigger sympathetic vasoconstriction and activation of vasoprotective mechanisms, resulting in changes in cerebral vascular structure and cognitive function. It is also believed that oxidative stress can damage mitochondrial oxidative phosphorylation, leading to synaptic damage [31]. Moreover, sleep fragmentation is considered another potential mechanism for memory impairment, which reduces the volume of gray matter in the total cortex, lateral orbitofrontal cortex, and suborbital frontal gyrus cortex [文献]. Other studies suggest that sleep deprivation‐induced damage underlies cognitive deficits and impairs vigilant attention, which requires rest or prolonged sleep before recovery [32]. OSA may activate microglia to produce a range of cytokines (IL‐1β, IL‐6, and TNF‐α), thus triggering central inflammatory responses, exacerbating inflammatory damage to the CNS, and impairing cognitive function [33]. Cerebral IH may convert astrocytes from A2 (protective) to A1 (injurious) via NLRP3 immunoenzymes, resulting in hippocampal neuronal disorders and cognitive decline [34].

#### 4.1.2 Association Between OSA and Anxiety and Possible Mechanisms

In the current study, the prevalence of anxiety symptoms was significantly higher in the OSA group than in the non‐OSA group (58.5% vs. 29.2%), emphasizing the need for a comprehensive assessment and management of patients with OSA. Similarly, a previous study documents a high prevalence of depression (35%) and anxiety symptoms (32%) in patients with obstructive sleep apnea syndrome [35]. The potential explanation may lie in the fact that OSA is often associated with symptoms such as fatigue and mood disorders, and depression and anxiety are more common in patients with severe OSA than in those with non‐severe OSA [36]. However, many studies of the association between OSA severity and psychiatric symptoms have produced inconsistent results [37]. In our study, OSA severity was not associated with depression, which is consistent with several other studies [38, 39, 40, 41, 42]. Meanwhile, most clinicians do not routinely consider mood problems such as depression or anxiety when treating patients with OSA, leading to a delayed diagnosis [43]. The deficiency in this aspect may be improved with the increased awareness of the association between these psychiatric disorders and OSA and the appropriate use of assessment tools. We recommend standardizing the use of the hospital anxiety and depression scale for patients with OSA, especially in sleep centers, to detect any depressive or anxiety‐related disorders that may exacerbate clinical symptoms and compromise the efficacy of the treatments.

### 4.2 OSA and EEG Microstates

EEG microstates are spatially coherent patterns of spontaneous neural activity in the brain on a millisecond time scale, reflecting the functional state of the large‐scale neural networks of the brain. Studies have shown that EEG microstates can be categorized into four main types: A, B, C, and D, each of which is associated with specific cognitive functions and neuropsychiatric disorders. In our study, the OSA group reported more microstate As and a significantly higher rate of B to A microstate conversion than the non‐OSA group, which is partially consistent with a previous finding that the coverage of the A and B microstates may increase with the aggravation of cognitive dysfunction and that of the C and D microstates may decrease in a gradient‐like manner [44]. In patients with OSA, an increase in EEG microstate A may be an adaptive or compensatory response of the brain to adverse stimuli, such as sleep apnea disorders, or it may be a manifestation of impaired brain function [45]. Such an increase may interfere with the brain's normal regulation of the attention‐related network, leading to inattention and distractibility.

The possible mechanism is that patients with OSA often experience repeated upper airway obstruction during sleep, leading to sleep fragmentation and frequent transitions from states such as deep sleep to light sleep or wakefulness. This alteration in sleep architecture affects the normal rhythm of EEG activity and may make EEG microstates more susceptible to transitions [45]. OSA causes intermittent hypoxia and retention of carbon dioxide, which can lead to compromised metabolism and function of neurons in the brain, and hypoxia may alter neuronal excitability, making transitions from EEG microstate B to A more likely to occur in an attempt to maintain the normal function of the brain [46]. The higher conversion rate may be a compensatory mechanism for the brain in the face of adverse effects caused by OSA.

### 4.3 OSA and EEG Alpha Peak Frequency

In this study, the comparative analysis revealed significantly lower Delta and Theta power at the F7‐AV position in the OSA group than in the non‐OSA group, which may reflect less active slow wave activity in the prefrontal region in the OSA group. As Delta and Theta waves are usually correlated with deep sleep (slow‐wave sleep), the lower Delta and Theta power in the OSA group may indicate their poorer quality of deep sleep and less stable and restorative sleep [47]. Given that prefrontal regions are closely related to higher brain functions such as cognitive function, emotion regulation, and decision making, and that the OSA group reported less active slow wave activity in the prefrontal region, it follows that the OSA patients may have poorer cognitive function and emotion regulation, which may be attributed to sleep fragmentation and hypoxia. The stronger slow‐wave activity in the non‐OSA group may reflect their better neurological recovery and regulation during sleep [48]. We further explored the correlation of Delta and Theta power at the F7‐AV position with PSG sleep staging, which showed a positive correlation of Delta and Theta power at the F7‐AV position with stage N2 (*r* = 0.226, *p* = 0.06; *r* = 0.322, *p* = 0.02, respectively), and no significant correlation with stage N3. Further in‐depth research is awaited to elucidate the observed disparity.

Moreover, the correlation analysis revealed that AHI was negatively correlated with the FZ‐AV Beta1 frequency in EEG alpha peak frequency (*r* = −0.346), and positively correlated with the P4‐AV Theta frequency (*r* = 0.26) and the O1‐AV Beta2 frequency (*r* = 0.257). As Beta1 frequency is usually correlated with alertness and cognitive activity, the negative correlation suggests that OSA may negatively affect the alertness or cognitive function of the brain. Since Theta band is usually associated with sleep and relaxation while Beta2 band may be associated with anxiety or hypervigilance, the increase in the AHI indicates a disrupted sleep architecture, with more light sleep and less deep sleep. This may result in the failure of the brain of OSA patients to fully relax during sleep or anxiety‐like brain electrical activity. This is consistent with the current finding that the prevalence of anxiety symptoms is significantly higher in the OSA group than in the non‐OSA group, which is likely the mechanism underlying the OSA‐induced alterations in the EEG microstates.

## 5 Conclusion

The current study demonstrates that OSA may affect sleep quality and cognitive function by influencing EEG activity and microstates, which may be a potential EEG network mechanism underlying the OSA‐induced cognitive impairment. However, this finding awaits further verification with more study subjects and imaging alterations. Given the importance of specific EEG parameters in the assessment and diagnosis of OSA, the monitoring of the EEG microstates and the α‐peak frequency may provide new perspectives into the neurophysiological mechanisms of OSA and for the evaluation of the OSA severity and relevant therapeutic efficacy. Future studies could further explore the relationship between these parameters and OSA, as well as their potential role in disease progression and treatment response.

## 6 Limitations

Several limitations remain in the study. First, this study recruited a limited number of study subjects due to the strict standard PSG requirements and exclusion of unqualified data. Second, because of the low acceptance of PSG in the normal population, the homogeneous enrollment of patients from the neurological department may have some impact on the results. This study used a cross‐sectional design and only intercepted data at a specific time point for analysis, which could not reveal the dynamic association between OSA and cognitive impairment over time. A longitudinal study is needed to expand the sample size and recruit the normal population as the control group and observe the dynamic changes in cognitive functions and breathing‐related sleep conditions in cognitively impaired patients comorbid with OSA.

## Conflicts of Interest

The authors declare no conflicts of interest.

## Data Availability

The data that support the findings of this study are available from the corresponding author upon reasonable request.
